# Topology-Stress-Based Wormhole Attack Defense for Power Wireless Sensor Networks with UWB Physical-Layer Awareness

**DOI:** 10.3390/s26134141

**Published:** 2026-07-01

**Authors:** Kaiyun Wen, Fan Li, Fangming Deng, Zhen Wang

**Affiliations:** 1 School of Electrical and Automation Engineering, East China Jiaotong University, Nanchang 330013, China; 2 School of Public Security, Ningxia Police Vocational College, Yinchuan 750021, China; 3 Research Institute of State Grid Jiangsu Electric Power Co., Ltd., Nanjing 211103, China

**Keywords:** power wireless sensor networks, ultra-wideband, wormhole attack, physical-layer features, trust evaluation, topology security

## Abstract

Power wireless sensor networks (PWSNs) provide essential field-level sensing and communication support for smart grids, where topology authenticity directly affects communication reliability and network operation. However, wormhole attacks can forge false adjacency relationships through low-latency tunnels, thereby disrupting topology consistency and misleading routing decisions. In practical power environments, metallic obstruction, multipath reflection, and non-line-of-sight (NLOS) propagation may further cause normal-ranging anomalies to resemble attack-induced topology distortion, making reliable wormhole attack detection challenging. To address this issue, this paper proposes a topology-stress-based wormhole attack defense method with ultra-wideband (UWB) physical-layer awareness. The first-path power ratio and root-mean-square delay spread extracted from UWB channel impulse responses are used to evaluate link-ranging reliability and construct adaptive stiffness coefficients. Local backbone links are modeled as virtual springs, and a topology stress indicator is derived from the residual deformation after potential-energy minimization to quantify the geometric inconsistency caused by forged adjacency relationships. Furthermore, a Beta-based temporal evidence fusion mechanism is introduced to support graded node access decisions and improve decision stability. Simulation and hardware validation results demonstrate that the proposed method effectively suppresses NLOS-induced false alarms while maintaining high sensitivity to wormhole attacks. Compared with representative baseline methods, it achieves more stable detection performance under increasing ranging errors and different attack intensities. Hardware experiments further show that topology stress can clearly distinguish normal links, NLOS-affected links, and forged wormhole links, confirming its effectiveness for topology-authenticity verification in power wireless sensor networks.

## 1. Introduction

Power wireless sensor networks (PWSNs) are important infrastructures for field sensing, condition monitoring, and data transmission in smart grids and have been widely deployed in substations, distribution rooms, cable tunnels, and other power facilities [1]. Compared with conventional wired monitoring systems, wireless sensor networks offer greater deployment flexibility, easier scalability, and lower retrofit costs, enabling continuous acquisition and multi-hop transmission of equipment-state information. However, the abundance of metallic equipment, complex spatial structures, and strong reflection environments in power systems poses significant challenges to topology construction and maintenance, making network topology more vulnerable to topology-deception attacks [2]. Among various security threats, wormhole attacks can forge adjacency relationships between distant nodes through low-latency private links, thereby compromising topology authenticity and affecting routing establishment, link maintenance, and data transmission reliability. Therefore, accurately identifying forged adjacency relationships caused by wormhole attacks and safeguarding topology authenticity have become important research issues for the secure and reliable operation of power wireless sensor networks.

Wormhole attacks are a typical form of topology-deception attack. In a wormhole attack, two colluding malicious nodes establish a low-latency private link and rapidly forward packets between distant network regions, causing legitimate nodes to mistakenly regard remote nodes as direct neighbors. Unlike data tampering, identity spoofing, or denial-of-service attacks, wormhole attacks do not require breaking encryption mechanisms or compromising a large number of network nodes. Instead, they manipulate the neighbor-discovery and topology-formation processes to create logical connectivity relationships that are inconsistent with the actual physical topology. As a result, wormhole attacks may mislead routing establishment, disrupt link maintenance, and further affect data aggregation, topology control, and network coordination, making them one of the most threatening security attacks in wireless sensor networks [3]. Considerable research efforts have been devoted to wormhole attack detection and defense. Early studies mainly relied on temporal constraints, geographical constraints, and neighbor-verification mechanisms to prevent the establishment of forged links. For example, Packet Leashes restrict packet propagation through temporal and spatial constraints, while LiteWorp identifies suspicious nodes through secure neighbor discovery and local monitoring mechanisms [4]. Subsequently, researchers investigated topology-based and geometry-consistency-based approaches, which detect wormhole attacks by analyzing topology reconstruction results, connectivity structures, and distance constraints [5,6]. More recently, with the development of artificial intelligence and Internet-of-Things security technologies, AI-based reviews, machine learning, federated learning, and trust-based approaches have also been introduced to improve anomaly detection capability in complex network environments [7,8,9,10]. Overall, existing wormhole defense methods mainly identify forged adjacency relationships from the perspectives of time, distance, topology structure, and node behavior, providing an important foundation for topology security in wireless sensor networks.

Despite the considerable progress achieved in wormhole attack detection, applying existing methods to power wireless sensor networks remains challenging. In typical power scenarios such as substations and distribution rooms, dense metallic equipment and complex spatial structures often result in non-line-of-sight (NLOS) propagation and severe multipath effects [11,12]. Consequently, legitimate links may also exhibit large ranging errors or unstable link-quality measurements, causing them to resemble attack-affected links. For methods that rely on distance measurements or topology relationships, environmental ranging errors may be misinterpreted as topology anomalies caused by wormhole attacks. Conversely, for methods based on communication behavior or historical statistics, wormhole links may still exhibit apparently good communication quality under certain conditions, thereby reducing detection effectiveness. Therefore, relying solely on ranging observations, fixed decision thresholds, or communication-layer information makes it difficult to achieve both low false-alarm rates and high detection accuracy in complex power environments. Distinguishing topology anomalies caused by environmental interference from forged adjacency relationships introduced by wormhole attacks remains a key challenge in power wireless sensor networks.

Ultra-wideband (UWB) technology offers a promising solution to the above challenges. Compared with communication-layer indicators such as RSSI and LQI, UWB not only enables high-accuracy ranging but also provides physical-layer information from channel impulse responses (CIRs) that reflect propagation conditions. CIR-based features have been widely used for non-line-of-sight (NLOS) identification and ranging error mitigation [13,14,15], providing stronger evidence for link reliability assessment. However, most existing studies focus on improving localization accuracy or compensating for ranging errors, while relatively little attention has been paid to exploiting UWB physical-layer information for analyzing topology anomalies caused by wormhole attacks. In particular, effective approaches for validating adjacency authenticity and distinguishing environmental interference from malicious topology forgery in complex power environments remain limited.

To address the challenge of distinguishing environmental interference from wormhole attacks in complex power environments, this paper proposes a wormhole defense method that integrates UWB physical-layer awareness with topology-stress analysis. The proposed method utilizes UWB channel impulse response features to assess link-ranging reliability and incorporates this information into local topology constraint modeling. Topology stress analysis is then employed to quantify the geometric inconsistency caused by forged adjacency relationships. Finally, a temporal trust-fusion mechanism is introduced to support dynamic node access decisions, thereby improving the accuracy and robustness of wormhole attack detection under complex propagation conditions.

The main contributions of this paper are summarized as follows:

(1) A UWB channel-impulse-response (CIR) feature-based adaptive stiffness modeling method is proposed to associate link propagation conditions with the reliability of distance constraints, providing trustworthy topology constraints in complex environments.
(2) A topology-stress-analysis method based on a virtual spring model is developed to identify forged adjacency relationships caused by wormhole attacks through characterization of local geometric constraint conflicts, without requiring global absolute location information.
(3) A Beta-based temporal evidence fusion mechanism is designed to convert consecutive observations into node trust evaluations, enabling graded defense strategies including normal access, restricted access, and access rejection.

The remainder of this paper is organized as follows. Section 2 reviews the related work on wormhole attack detection and topology security in wireless sensor networks. Section 3 presents the network model, attack model, and problem formulation. Section 4 describes the proposed wormhole attack defense method based on topology stress analysis. Section 5 provides experimental validation and result analysis. Finally, Section 6 concludes the paper.

## 2. Related Work

### 2.1. Wormhole Attack Detection in Wireless Sensor Networks

Wormhole attacks are among the earliest topology-deception attacks studied in wireless multi-hop networks. Hu et al. [3] proposed the Packet Leashes method, which limits the possible physical propagation range of packets through temporal and spatial constraints, laying the foundation for subsequent wormhole attack detection methods. Khalil et al. proposed LiteWorp, which identifies wormhole nodes through secure two-hop neighbor discovery and local monitoring [4]. Maheshwari et al. further demonstrated that wormhole-induced abnormal substructures can be detected using only network connectivity information [6]. These studies explain the basic characteristics of wormhole attacks from the perspectives of time constraints, neighbor relationships, and connectivity structures. However, most of these methods rely on relatively ideal time synchronization, reliable neighbor monitoring, or stable network connectivity.

In recent years, machine learning, deep learning, and trust mechanisms have been increasingly incorporated into wormhole attack detection. Hanif et al. reviewed artificial-intelligence-based methods for detecting wormhole attacks in wireless sensor networks. They noted that learning-based methods can extract attack patterns from complex communication features, though they depend on training data and scenario consistency [7]. Alghamdi and Bellaiche applied cascaded federated deep learning to detect wormhole attacks in IoT networks, enabling anomaly identification while preserving node-side data privacy [8]. Alshehri [9] analyzed node connectivity features using machine learning in hybrid IoT and WSN scenarios to detect and mitigate wormhole attacks. Zhukabayeva et al. further proposed a real-time detection and response mechanism for wormhole and sinkhole attacks in WSNs [10]. These studies enhance the intelligence and adaptability of wormhole attack detection. However, most of them still rely mainly on network traffic, routing behavior, or connectivity features as inputs, with limited use of wireless propagation conditions and physical-layer ranging reliability.

Therefore, existing network-layer and learning-based methods still fail to adequately account for the propagation uncertainty caused by metallic reflections, multipath propagation, and NLOS conditions in power environments.

### 2.2. Geometry-Based Wormhole Detection and Topology Verification Methods

Geometry-based methods constitute an important category of wormhole attack defense approaches. The essence of a wormhole attack is to compress the logical distance between distant nodes through a low-latency tunnel, causing the network to form adjacency structures that are inconsistent with actual spatial relationships. Based on this observation, some studies have developed wormhole detection methods from the perspectives of distance constraints and topology geometry. Wang and Bhargava proposed MDS-VOW, which reconstructs the sensor network topology using multidimensional scaling and locates wormhole regions by observing bending and folding phenomena in the reconstructed topology [5]. This method does not rely on specialized encryption mechanisms and can reflect the geometric distortion caused by wormhole attacks through topological morphology. Subsequent studies have also identified wormhole attacks using localization constraints, neighbor distance relationships, path hop counts, and topology connectivity [6]. Yu et al. [16] studied secure localization in wireless sensor networks under wormhole attacks, demonstrating that position and distance consistency remain important indicators for wormhole defense.

Geometry-consistency-based detection methods usually assume that communication relationships among normal nodes should be broadly consistent with their physical spatial distribution [17]. In contrast, a wormhole attack artificially pulls remote nodes into the local neighborhood, distorting the local topology. The advantage of such methods is that they characterize wormhole attacks from a structural perspective rather than relying solely on identity authentication or traffic statistics. However, in complex power environments, distance observations are not always reliable. Ordinary NLOS links may also introduce large ranging errors and increase geometric residuals. Conversely, if a wormhole link is relayed through a nearby malicious node, the local physical signal may still exhibit good link quality. In this case, relying solely on distance thresholds or topology residuals makes it difficult to distinguish NLOS-induced errors from structural distortion caused by attacks.

Therefore, recent studies have increasingly focused on integrating geometry-based wormhole detection with link-reliability assessment to distinguish topology inconsistency caused by environmental errors from forged adjacency relationships [18].

### 2.3. UWB Physical-Layer Awareness and Trust-Based Wormhole Defense

Physical-layer-aware methods have recently emerged as a promising direction for wormhole attack defense. Among them, UWB technology provides a more direct basis for evaluating wireless link reliability. UWB technology offers high temporal resolution, enabling time-of-flight-based distance estimation and extraction of features such as first-path energy, received power, multipath distribution, and delay spread from channel impulse responses (CIRs). IEEE 802.15.4z enhances the UWB physical layer and related ranging mechanisms, providing a standardized foundation for high-precision ranging and secure distance verification [19].

In recent years, UWB NLOS identification and ranging error mitigation have received increasing attention. Yang et al. reduced ranging errors by using channel-impulse-response feature parameters and a two-step NLOS identification method [11]. Coene et al. corrected UWB ranging errors by combining channel features with location-related information [12]. References [13,14,15] studied UWB LOS/NLOS identification from the perspectives of machine learning classification, human-body shadowing detection, and lightweight detection, respectively. These studies show that UWB CIR features can effectively reflect first-path attenuation, multipath spread, and changes in the propagation environment. Recent survey studies have further highlighted the importance of NLOS identification and ranging reliability assessment in complex UWB localization environments [20]. Beyond ranging accuracy, recent studies have also investigated the security of UWB ranging systems. Security analyses of IEEE 802.15.4z and practical attack experiments have demonstrated that physical-layer attacks may still affect ranging authenticity even under enhanced ranging protocols [21,22,23]. These findings indicate that accurate ranging does not necessarily guarantee trustworthy topology relationships, highlighting the importance of combining physical-layer observations with topology-security mechanisms.

Trust-based approaches provide another important line of defense for wireless sensor network security. The Beta reputation system and the Reputation-based Framework for Sensor Networks (RFSN) convert consecutive observations into probabilistic trust evaluations and support long-term security decision-making [24,25]. Recent evaluations have further analyzed the applicability of trust management frameworks in wireless sensor networks and confirmed their effectiveness in improving decision robustness under uncertain environments [26].

However, most existing trust models rely primarily on communication behavior, packet-forwarding statistics, or historical interactions, with limited consideration of topology distortion caused by wormhole attacks. For wormhole attack defense, a critical question remains unanswered: whether the adjacency relationship established by ranging constraints is authentic. A wormhole attack creates forged logical neighbors through a low-latency tunnel, thereby disrupting the consistency between the local topology and the actual physical space. Consequently, relying solely on communication behavior, historical interactions, or ranging reliability remains insufficient to identify structural topology distortion caused by malicious topology forgery.

In summary, UWB physical-layer features provide valuable information for assessing link reliability, while trust mechanisms improve the robustness of security decisions. Nevertheless, existing studies mainly focus on localization enhancement, NLOS identification, secure ranging, or behavior-based trust evaluation, while paying limited attention to wormhole-induced topology distortion and forged adjacency relationships. Bridging this gap remains a key challenge for wormhole defense in power wireless sensor networks.

## 3. System Model and Problem Formulation

### 3.1. Network and Attack Model

Consider a power wireless sensor network (PWSN) deployed in substations or distribution rooms consisting of ordinary sensing nodes, aggregation terminal nodes, and a base station. Sensing nodes collect equipment-state information and upload it to nearby aggregation terminals, which perform intra-cluster aggregation and multi-hop backbone forwarding to the base station. Since adjacency relationships among aggregation terminals determine backbone routing and data-backhaul reliability, this paper focuses on the topology security of the aggregation-terminal backbone network.

The backbone network is represented as a graph *G* = (*V*, *E*), where *V* = {*H*_0_, *H*_1_, …, *H_M_*} denotes the set of base station and aggregation terminal nodes, H_0 is the base station node, and *E* denotes the set of backbone links that satisfy the communication and neighbor verification conditions. Under normal conditions, the backbone links should be broadly consistent with the actual spatial distribution of nodes. That is, only nodes that are physically close and have satisfactory link quality can establish direct adjacency relationships.

**Figure 1:**
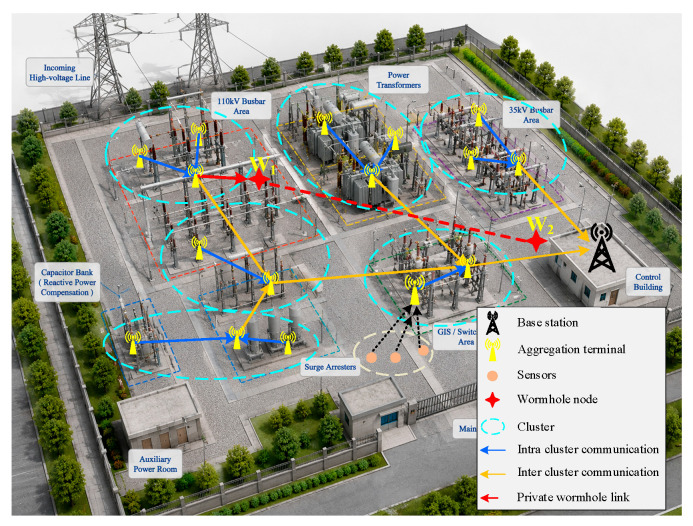
Wormhole attack scenario in a clustered power wireless sensor network deployed in a substation.

As shown in Figure 1, a wormhole attack creates forged adjacency relationships between distant nodes through two colluding malicious nodes, W_1_ and W_2_. These two nodes are deployed at distant locations in the network and form a wormhole tunnel through a low-latency private link. When W_2_ intercepts neighbor-discovery packets, routing broadcasts, or control messages from a remote area, it can rapidly forward them through the tunnel to W_1_, which then rebroadcasts them in the local area. As a result, legitimate nodes may mistakenly regard distant nodes as one-hop neighbors, leading to cross-region links that do not actually exist. The goal of the proposed method is to identify such forged adjacency relationships caused by wormhole attacks in the absence of global absolute coordinates and under complex propagation conditions, thereby providing a basis for subsequent decisions on abnormal links and suspicious nodes. It should be noted that the proposed method does not rely on prior knowledge of wormhole attack occurrence probabilities. Instead, wormhole attacks are treated as unknown network anomalies, and forged adjacency relationships are identified directly through physical-layer observations and topology-consistency analysis.

In conventional wireless sensor networks, forged adjacency relationships can often be identified through distance constraints or topology-consistency analysis. However, in power environments, non-line-of-sight (NLOS) propagation and multipath effects may introduce ranging deviations and local topology anomalies. As a result, topology distortion caused by environmental factors may exhibit distortion patterns similar to those caused by wormhole attacks. Therefore, distinguishing environment-induced topology anomalies from forged adjacency relationships remains a key challenge for wormhole defense in power wireless sensor networks.

### 3.2. Local Geometric Consistency Model

In the absence of global absolute coordinates, a local relative coordinate system is adopted to describe the geometric relationships within the neighborhood of the node under verification. Let the node under verification be denoted by *u*, and let *N_u_* represent its set of valid verification neighbors. In the local coordinate system established by reference nodes that have completed access authentication and remain in a stable state, the position of any neighboring node *H_j_* is expressed as

(1) $$p_{j}={\left[x_{j},y_{j}\right]}^{T}$$

For a legitimate link, the compensated distance estimate obtained from UWB ranging should be broadly consistent with the Euclidean distance in the local relative coordinate system, and the ranging uncertainty provides an upper bound for the deviation. The local geometric consistency deviation between node *u* and its neighboring node *H_j_* is defined as

(2) $$\Delta_{uj}={\left\|p_{j}\right\|}_{2}-{\hat{d}}_{uj}$$

where *p_u_* is the local position of the node under verification *u*, *p_j_* is the local position of the neighboring node *H_j_*, and ${\hat{d}}_{uj}$ is the compensated distance estimate of link (*u*, *j*).

Under normal conditions, the geometric deviation of the link should satisfy

(3) $$\left|\Delta_{uj}\right|=\left|{\left\|p_{j}\right\|}_{2}-{\hat{d}}_{uj}\right|\leq \lambda {\hat{\sigma }}_{uj}^{2}$$

where ${\hat{\sigma }}_{uj}^{2}$ denotes the ranging uncertainty of link (*u*, *j*), and *λ* is the confidence constraint coefficient. Equation (3) does not require the local topology to form a strictly rigid geometric structure. Instead, it requires the compensated ranging result to remain broadly consistent with the local geometric relationship. When a wormhole attack forges a long-distance adjacency relationship, the abnormal link introduces a distance constraint that is difficult to reconcile in the local geometric space, causing the geometric consistency deviation to increase significantly.

### 3.3. Distance Observation Model with Physical-Layer Priors

In power field environments, distance observations obtained by aggregation terminal nodes are affected by propagation conditions, measurement noise, and attack behavior. For legitimate links, the compensated distance estimate can be modeled as an observation that fluctuates slightly around the true distance. Under a wormhole attack, however, the distance observation is further affected by the attack-induced bias introduced by the forged adjacency relationship. Therefore, the distance observation used for local topology discrimination can be expressed as

(4) $${\hat{d}}_{ij}^{c}=d_{ij}+(1-\gamma_{ij})\Delta_{ij}^{atk}+\xi_{ij}$$

where *d_ij_* is the true Euclidean distance between nodes *H_i_* and *H_j_*, and $\Delta_{ij}^{atk}$ is the attack-induced bias introduced by the wormhole attack. $\gamma_{ij}\in \{0,1\}$ is the link-legitimacy indicator, where *γ* = 1 denotes a legitimate link and *γ* = 0 denotes a wormhole attack link. $\xi_{ij}$ is the residual disturbance after compensation, whose dispersion is characterized by the estimated ranging uncertainty.

For a wormhole link, the attacker replays packets from a remote area into the local area through the private tunnel between the nearby malicious node and the remote colluding node. In this case, a legitimate node may incorrectly regard the remote node *H_j_* as a direct neighbor. However, the physical-layer observation is closer to the propagation process between the legitimate node and the nearby malicious node W_1_. Therefore, the attack bias usually appears as a distance-compression effect and can be approximated as

(5) $$\Delta_{ij}^{atk}\approx d_{iW_{1}}-d_{ij}<0$$

where W_1_ denotes the nearby malicious relay node close to the node under verification *H_i_*. Since *d_iW1_* ≪ *d_ij_* usually holds, the wormhole attack causes the compensated distance observation to be abnormally compressed.

## 4. Wormhole Attack Defense Method Based on Topology Stress Analysis

### 4.1. UWB Physical-Layer Feature-Aware Adaptive Stiffness Modeling

In the virtual spring model, link stiffness is used to describe the influence of distance constraints on the local topology potential energy. If a fixed stiffness is assigned to all links, ranging deviations caused by NLOS propagation, multipath effects, and instantaneous interference may be misinterpreted as attack-induced anomalies. To reduce false alarms in complex power environments, this paper adaptively adjusts link stiffness using UWB physical-layer features.

From a statistical perspective, a smaller ranging error variance indicates a more reliable distance constraint, and thus a larger stiffness value should be assigned. Conversely, a larger ranging error variance indicates a less reliable distance constraint, and its influence on the potential energy function should be reduced. Therefore, link stiffness can be set inversely proportional to the ranging error variance:

(6) $$k_{uj}=\frac{1}{\sigma_{uj}^{2}}$$

where $\sigma_{uj}^{2}$ is the ranging error variance of link (*u*, *j*). Since the ranging error variance is difficult to obtain directly in practical deployments, UWB channel impulse response features are used for approximate characterization.

(7) $$k_{uj}\approx c_{0}\cdot {\left(\frac{P_{FP}}{P_{RX}}\right)}^{\lambda_{1}}\cdot {\left(\frac{1}{1+\frac{\tau_{rms}}{\tau_{0}}}\right)}^{\lambda_{2}}$$

where *P_FP_* and *P_RX_* denote the first-path signal power and the total received power, respectively. When their ratio approaches 1, the direct-path component is dominant, indicating higher ranging reliability and thus a larger stiffness coefficient *k_uj_*. $\tau_{rms}$ and $\tau_{0}$ denote the root-mean-square delay spread and the normalized reference time constant, respectively. As $\tau_{rms}$ increases, the influence of multipath reflection becomes stronger, the ranging error increases, and the stiffness coefficient *k_uj_* decreases accordingly. Here, *c*_0_, *λ*_1_, and *λ*_2_ are empirical parameters.

### 4.2. Topology Stress Construction Based on the Virtual Spring Model

Wormhole attacks establish forged adjacency relationships between physically distant nodes, causing inconsistency between the network topology and the actual spatial distribution of nodes. Once such forged links are introduced into the local topology, the original distance constraints among neighboring nodes become difficult to satisfy simultaneously. In a legitimate topology, neighboring links generally exhibit good geometric consistency and can reach a coordinated equilibrium state. In contrast, wormhole-induced forged links introduce conflicting geometric constraints that distort the local topology structure. To quantify this inconsistency caused by forged adjacency relationships, a topology stress indicator is constructed based on a virtual spring model and used to characterize the degree of local topology distortion. To characterize wormhole-induced local geometric distortion, aggregation terminals and communication links are modeled as mass points and virtual springs, respectively, with the compensated UWB ranging value used as the spring natural length. A link contributes negligible potential energy when its geometric distance is consistent with the ranging constraint, whereas a large inconsistency causes stretching or compression and accumulates local potential energy.

Let *u* denote the node under verification and $N_{u}$ denote its valid verification neighbor set. In the local relative coordinate system, the position of the node under verification is denoted by *p_u_*, and the position of neighboring node *H_j_* is denoted by *p_j_*. The local potential energy function of node *u* is defined as the sum of the elastic potential energy of the links between node *u* and all its verification neighbors:

(8) $$E_{u}(p_{u})=\sum_{j\in N_{u}}\frac{1}{2}k_{uj}{\left(\|p_{u}-p_{j}{\|}_{2}-{\hat{d}}_{uj}^{c}\right)}^{2}$$

where *k_uj_* is the virtual stiffness coefficient of link (*u*, *j*), which describes the strength of the geometric constraint imposed by the link, and $N_{u}$ is the valid verification neighbor set of node *u*. When the local geometric distance equals the compensated distance observation, the potential energy of the corresponding link is zero. When a deviation exists between them, a larger deviation and a higher stiffness result in greater accumulated potential energy in the link.

To characterize the geometric equilibrium state of node *u* under the current local topology constraints, the position of node *u* is estimated by minimizing the local potential energy function. This process can be formulated as a nonlinear unconstrained optimization problem:

(9) $$p_{u}=\arg\min_{p_{u}}\sum_{j\in N_{u}}\frac{1}{2}k_{uj}{\left(\|p_{u}-p_{j}{\|}_{2}-{\hat{d}}_{uj}^{c}\right)}^{2}$$

where $N_{u}$ is the valid verification neighbor set of node *u*, and ${\hat{d}}_{uj}^{c}$ is the compensated distance observation between node *u* and node *j*. The optimization problem is solved iteratively using momentum-based gradient descent. First, the negative gradient of the potential field with respect to the node coordinates is calculated, which represents the virtual resultant force acting on node *u* at its current position:

(10) $$F_{u}=-\nabla_{p_{u}}E_{u}=-\sum_{j\in N_{u}}k_{uj}({\left\|p_{u}-p_{j}\right\|}_{2}-{\hat{d}}_{uj}^{c})\cdot n_{uj}$$

where $n_{uj}=\frac{p_{u}-p_{j}}{\|p_{u}-p_{j}{\|}_{2}}$ is the unit direction vector from node *j* to node *u*. This resultant force reflects the overall deviation between the current position and the local distance constraints. The iterative update equations for the node coordinates are given as follows:

(11) $$v^{\left(t\right)}=\mu v^{\left(t-1\right)}+\eta F_{u}(p_{u}^{\left(t-1\right)})$$

(12) $$p_{u}^{\left(t\right)}=p_{u}^{\left(t-1\right)}+v^{\left(t\right)}$$

where v is the velocity vector, μ is the momentum factor, and η is the learning rate. When the norm of the resultant force falls below the preset convergence threshold or the maximum number of iterations is reached, the system is considered to have reached mechanical equilibrium. The corresponding coordinate is then taken as the local optimal position estimate of the node under the current topology constraints.

Ideally, after the system reaches equilibrium, each virtual spring should approach its natural length, and the local potential energy should remain at a low level. However, when a wormhole attack exists in the network, the forged link introduced by the attacker conflicts with the geometric constraints imposed by legitimate links, making the local topology difficult to satisfy simultaneously in Euclidean space. Consequently, even after the iterative optimization converges, noticeable stretching and compression effects may still remain, resulting in abnormal topology stress.

To quantify the anomaly caused by such topology distortion, a topology stress indicator is defined. For node *u* and its neighboring node *j*, the residual topology stress $\psi_{uj}$ on link (*u*, *j*) is defined as the product of the stiffness coefficient and the residual deformation:

(13) $$\psi_{uj}=k_{uj}\cdot \left|{\left\|p_{u}^{*}-p_{j}\right\|}_{2}-{\hat{d}}_{uj}^{c}\right|$$

where *k_uj_* is the adaptive stiffness coefficient of link (*u*, *j*), $p_{u}^{*}$ is the optimal position estimate of node *u* after the local potential energy reaches equilibrium, and ${\hat{d}}_{uj}^{c}$ is the compensated distance observation between node *u* and node *j*. This indicator reflects the degree of unresolved geometric constraint conflict on the link after equilibrium is achieved. A larger residual topology stress indicates that the link is less consistent with the local geometric structure. Furthermore, the aggregated topology stress indicator $\Psi_{u}$ of node *u* is defined as the normalized squared aggregation of the residual topology stresses within its neighborhood:

(14) $$\Psi_{u}=\frac{1}{\sum_{j\in N_{u}}k_{uj}}\sum_{j\in N_{u}}\psi_{uj}^{2}$$

For a legitimate topology, distance constraints among neighboring nodes are generally consistent, allowing the virtual spring system to converge to a relatively low topology stress level. In contrast, when a wormhole attack introduces forged adjacency relationships, some distance constraints become inconsistent with the surrounding geometric configuration, resulting in larger residual deformation and increased topology stress. Therefore, topology stress can serve as an effective indicator of local topology consistency. A higher topology stress value implies a greater likelihood of topology distortion within the local neighborhood. Based on this observation, the calculated topology stress is further incorporated into the subsequent trust-evaluation process to improve the accuracy and stability of wormhole attack detection.

### 4.3. Temporal Trust Fusion and Node Access Decision

A single topology stress observation may be affected by instantaneous channel fluctuations, ranging noise, and local environmental changes. To improve the stability of node access decisions, a Beta reputation model is introduced to convert the topology-stress sequence obtained from consecutive observations into a temporal evidence stream and dynamically evaluate the legitimacy of the node under verification.

The identity authenticity of the node under verification u is modeled as a Bernoulli random variable, and a Beta distribution is used to describe its legitimacy probability. The reputation probability density function of node u at time t is given by

(15) $$f(p|\alpha_{u}^{\left(t\right)},\beta_{u}^{\left(t\right)})=\frac{\Gamma (\alpha_{u}^{\left(t\right)}+\beta_{u}^{\left(t\right)})}{\Gamma (\alpha_{u}^{\left(t\right)})\Gamma (\beta_{u}^{\left(t\right)})}p^{\alpha_{u}^{\left(t\right)}-1}{\left(1-p\right)}^{\beta_{u}^{\left(t\right)}-1}$$

where p∈[0,1] denotes the probability that the node behavior is legitimate, and Γ(⋅) is the Gamma function. $\alpha_{u}^{\left(t\right)}$ and $\beta_{u}^{\left(t\right)}$ are the positive and negative evidence factors at time *t*, respectively. Based on this probability distribution, the expected trust value of node *u* is defined as:

(16) $$T_{u}^{\left(t\right)}=E[p]=\int_{0}^{1}p\cdot f(p|\alpha_{u}^{\left(t\right)},\beta_{u}^{\left(t\right)})dp=\frac{\alpha_{u}^{\left(t\right)}}{\alpha_{u}^{\left(t\right)}+\beta_{u}^{\left(t\right)}}$$

This expected value is used as the quantitative basis for the final decision. During initialization, $\alpha_{u}^{\left(0\right)}=\beta_{u}^{\left(0\right)}=1$, corresponding to a uniform prior distribution, which indicates that no prior bias is assigned to the node under verification. Let *Ψ_th_* denote the safety threshold of the aggregated topology stress. When the *t*-th observation satisfies *Ψ_u_*(*t*) < *Ψ*_th_, the current local topology is considered to satisfy the geometric coordination constraint, and the system accumulates positive evidence.

(17) $$\left\{\begin{array}{l} \alpha_{u}^{\left(t\right)}=\alpha_{u}^{\left(t-1\right)}+1\\ \beta_{u}^{\left(t\right)}=\beta_{u}^{\left(t-1\right)} \end{array}\right.$$

This update rule allows the trust value to increase smoothly and prevents it from rising too rapidly due to a single normal observation. When *Ψ_u_*(*t*) ≥ *Ψ_th_*, the local topology is considered to contain a potential anomaly. To distinguish slight environmental disturbances from significant geometric conflicts caused by wormhole attacks, an exponential negative-evidence increment is introduced.

(18) $$\Delta \beta =\exp\left(\eta \cdot \frac{\Psi_{u}^{\left(t\right)}-\Psi_{th}}{\Psi_{th}}\right)$$

where *η* is the sensitivity adjustment coefficient. The corresponding evidence-factor update rule is given as follows:

(19) $$\left\{\begin{array}{l} \alpha_{u}^{\left(t\right)}=\alpha_{u}^{\left(t-1\right)}\\ \beta_{u}^{\left(t\right)}=\beta_{u}^{\left(t-1\right)}+\Delta \beta \end{array}\right.$$

This mechanism adaptively adjusts the punishment strength according to the anomaly degree. For environmental disturbances that only slightly exceed the threshold, the negative-evidence increment remains small, and the trust value decreases slowly, reflecting a certain degree of fault tolerance. For wormhole attacks that significantly exceed the threshold, the negative-evidence increment increases rapidly, driving the negative evidence factor to grow quickly. As a result, the trust value drops rapidly, enabling fast identification of high-risk nodes.

**Figure 2:**
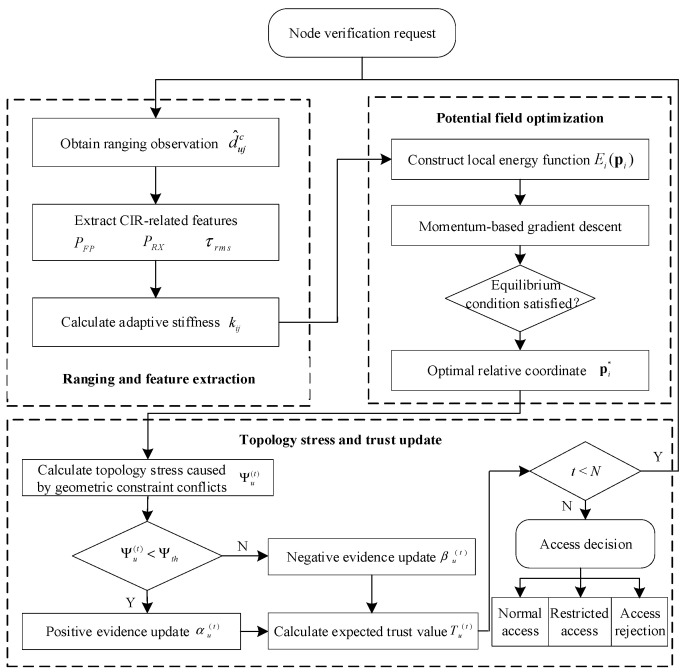
Overall procedure of the proposed wormhole attack defense mechanism based on topology stress analysis.

After *N* consecutive evaluations, the final expected trust value *T_u_* determines the access decision. A node is normally admitted if $T_{u}\geq T_{high}$, rejected and isolated if $T_{u}\leq T_{low}$, and granted restricted access if $T_{low}<T_{u}<T_{high}$. In the restricted-access state, the node can upload sensing data but cannot participate in backbone forwarding or critical cooperative tasks. The overall procedure of the topology-stress-based wormhole defense strategy is shown in Figure 2.

## 5. Simulation and Experimental Analysis

### 5.1. UWB Physical-Layer Awareness for Reliable Wormhole Defense

To verify the effectiveness of physical-layer prior parameter extraction and the adaptive stiffness model in the proposed wormhole attack defense mechanism, this paper first constructs an ultra-wideband channel model based on the IEEE 802.15.4a standard using the MATLAB R2023a simulation platform. The experiment primarily evaluates the accuracy of physical-layer feature extraction and the adaptive stiffness model’s ability to distinguish line-of-sight and non-line-of-sight links. The parameters listed in Table 1 are used to configure the IEEE 802.15.4a-based UWB channel model rather than additional parameters of the proposed defense algorithm. Specifically, the power-delay decay constant, the number of multipath components, the maximum relative delay, and the first-path normalized gain determine the multipath propagation characteristics of the simulated channel impulse responses. These simulated CIRs are subsequently used to extract the first-path power ratio and RMS delay spread, which serve as the physical-layer inputs for the adaptive stiffness model described in Section 4.1. The detailed parameter configuration is shown in Table 1.

**Figure 3:**
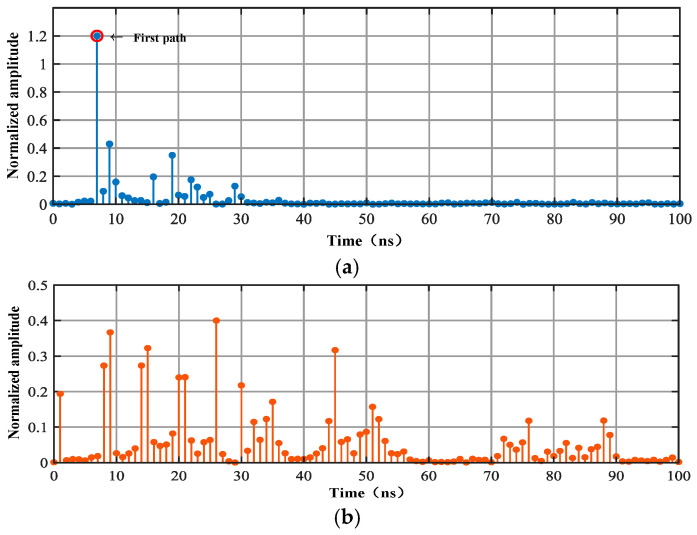
Comparison of UWB channel impulse response under LOS and NLOS. (**a**) CIR under LOS conditions. (**b**) CIR under NLOS conditions.

The CIR characteristics under LOS and NLOS scenarios are first analyzed to verify the effectiveness of the adaptive stiffness coefficient in quantifying link reliability. As shown in Figure 3a, the received energy in the LOS scenario is mainly concentrated in the first path, indicating dominant direct-path propagation and a high first-path power ratio. In contrast, Figure 3b shows that the NLOS signal energy is more dispersed over the delay domain, with a weakened first-path component and more pronounced multipath components. This observation confirms that NLOS propagation may introduce a positive bias into time-of-flight (ToF)-based ranging.

**Figure 4:**
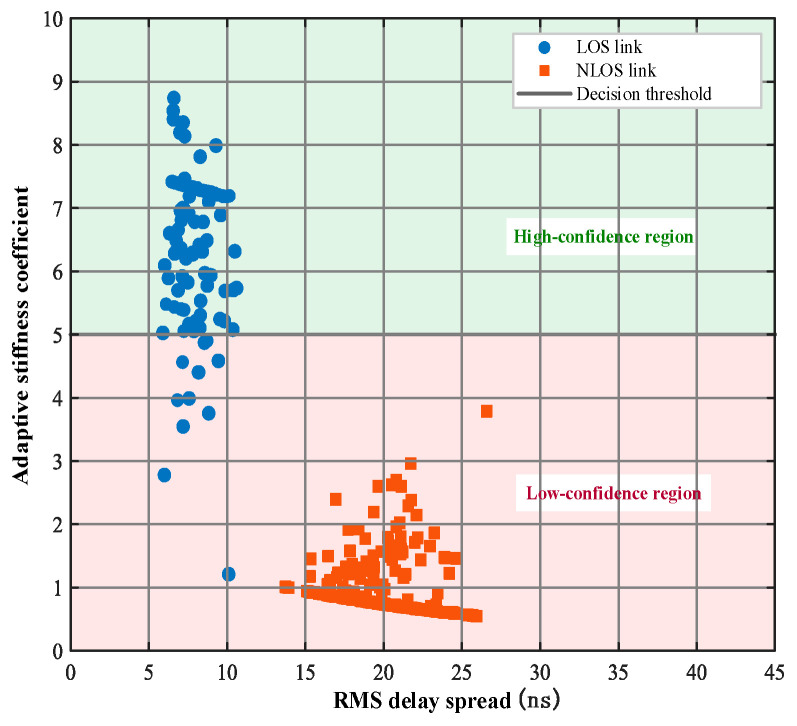
Distribution of stiffness coefficients under different channel scenarios.

Figure 4 further illustrates the relationship between the root-mean-square (RMS) delay spread and the adaptive stiffness coefficient over 400 LOS and NLOS channel samples. LOS samples are mainly distributed in the low-delay-spread and high-stiffness region, whereas NLOS samples exhibit larger delay spreads and lower stiffness values. The clear separation between the two distributions indicates that the proposed stiffness model can effectively distinguish link-ranging reliability based on UWB physical-layer features and provide adaptive constraint weights for subsequent topology stress analysis.

**Figure 5:**
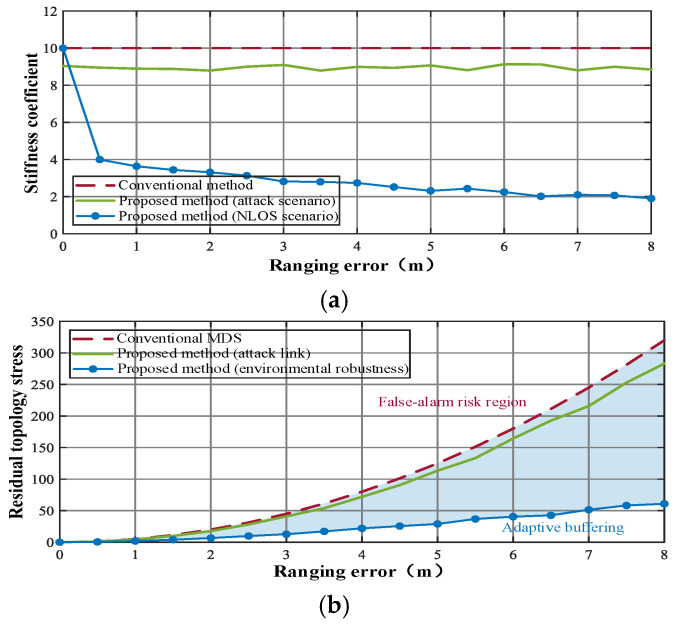
Differential response of adaptive stiffness model to ranging errors. (**a**) Stiffness coefficient response under different channel conditions. (**b**) Residual topology stress response under different error scenarios.The shaded area in (**b**) denotes the adaptive buffering region for environmentally induced ranging errors.

Figure 5 compares the fixed-stiffness method with the proposed adaptive stiffness model under different error scenarios. The fixed-stiffness method assigns identical weights to all links and therefore cannot distinguish NLOS-induced ranging errors from attack-induced anomalies. In contrast, the proposed method reduces the stiffness of unreliable NLOS links, thereby suppressing the influence of ordinary environmental errors on topology stress. When a wormhole attack occurs, the forged adjacency relationship introduces geometric conflicts that cannot be eliminated solely through stiffness adjustment, resulting in a significant increase in residual topology stress. Therefore, the adaptive stiffness model reduces false alarms under NLOS conditions while retaining sensitivity to wormhole attacks. These results demonstrate that the proposed model can effectively differentiate environmental ranging errors from attack-induced topology distortion, thereby providing a reliable basis for subsequent wormhole attack identification.

These results demonstrate that UWB physical-layer features contribute not only to ranging enhancement but also to reliability-aware topology analysis. By providing adaptive constraint weights for topology stress construction, the proposed method effectively suppresses NLOS-induced false alarms while preserving sensitivity to wormhole-induced topology distortion.

### 5.2. Potential Energy Evolution and Topology Stress Response Analysis

This section analyzes the local potential-energy evolution, topology-stress response, and temporal access-decision results under wormhole attack conditions through simulation experiments to verify the effectiveness of the proposed defense mechanism. A 50 m × 50 m local backbone network scenario is constructed, in which *N* = 20 routing nodes are randomly deployed. The communication radius is set to 20 m to maintain network connectivity. A malicious link is inserted between the core gateway *H*_0_ and the edge victim node *u*. The forged ranging value and the corresponding high stiffness coefficient are introduced to simulate a private communication tunnel with extremely low delay and high signal-to-noise ratio. The detailed simulation environment and key parameter settings are listed in Table 2.

**Figure 6:**
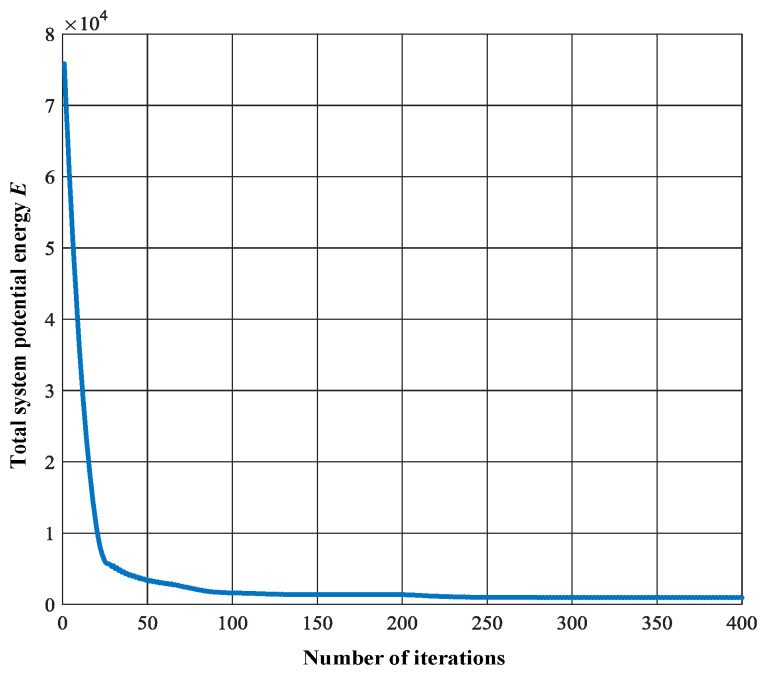
Evolution of total system potential energy during iterative optimization.

Figure 6 shows the variation in the total system potential energy *E* with the number of iterations when a wormhole attack is present. At the initial stage of the iteration process, the randomly initialized node positions exhibit large deviations from the distance constraints, resulting in a high total potential energy. As the gradient-descent iteration proceeds, the node positions are continuously adjusted toward the equilibrium state, and the potential energy decreases rapidly. After approximately 200 iterations, the total system potential energy becomes stable and converges to a nonzero residual-energy level. This indicates that the geometric constraints within the local topology cannot be simultaneously satisfied, and persistent geometric conflicts remain in the system.

**Figure 7:**
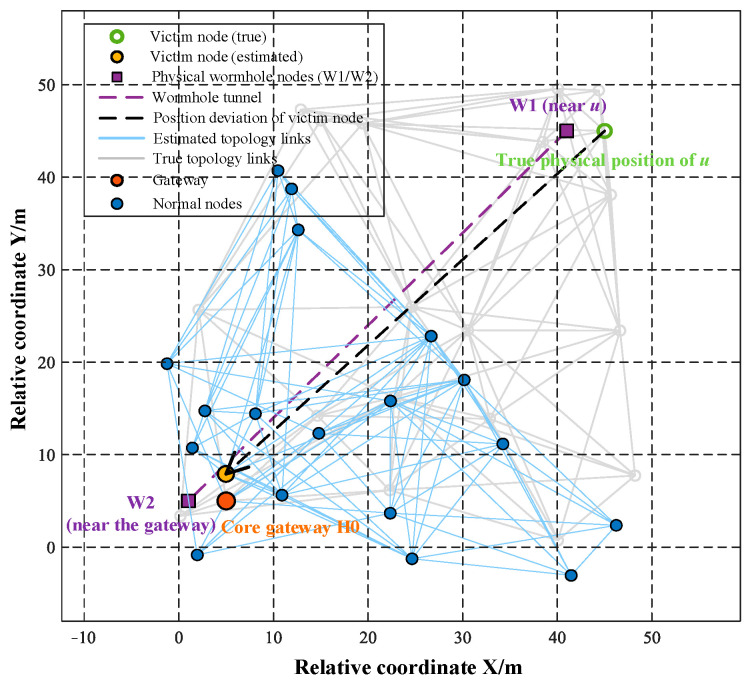
Topology folding caused by the wormhole attack.

Figure 7 illustrates the topology-folding phenomenon caused by a wormhole attack. Since the attacker forges a high-stiffness short link between the victim node and the core gateway, the local geometric constraints are significantly altered. During potential-energy minimization, the estimated position of the victim node shifts markedly toward the core gateway, causing the local topology that originally lies at the network edge to contract toward the gateway and resulting in a topology-folding structure. This phenomenon intuitively demonstrates that a wormhole attack not only changes the distance observation of a single link but also disrupts the consistency between the local topology structure and the actual spatial distribution.

**Figure 8:**
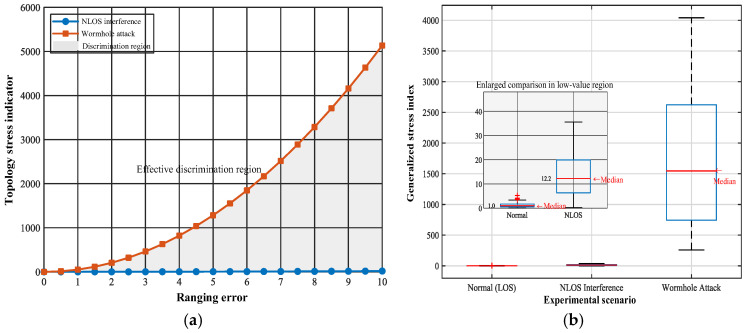
Topology stress responses under different scenarios: (**a**) Mean topology stress under different scenarios; (**b**) Statistical distribution of topology stress under different scenarios.

Figure 8 further confirms the discrimination capability of the topology-stress indicator. Although both NLOS interference and wormhole attacks introduce distance deviations, their stress responses differ significantly. The adaptive stiffness model suppresses links with large delay spread, keeping the topology-stress values under NLOS interference low and concentrated. In contrast, forged adjacency links introduce mutually incompatible geometric constraints, causing both the topology-stress value and its dispersion to increase markedly. These results indicate that the proposed indicator can effectively distinguish ordinary NLOS-induced ranging errors from structural topology anomalies caused by wormhole attacks.

The above results demonstrate that topology stress effectively captures the geometric inconsistency introduced by forged adjacency relationships. Compared with ordinary NLOS errors, wormhole attacks produce more severe topology distortion and consequently larger topology-stress values. Therefore, topology stress provides a direct and effective indicator for identifying wormhole-induced topology anomalies and forms the basis of the proposed defense mechanism.

### 5.3. Defense Performance Evaluation Under Different Error Levels and Attack Intensities

**Figure 9:**
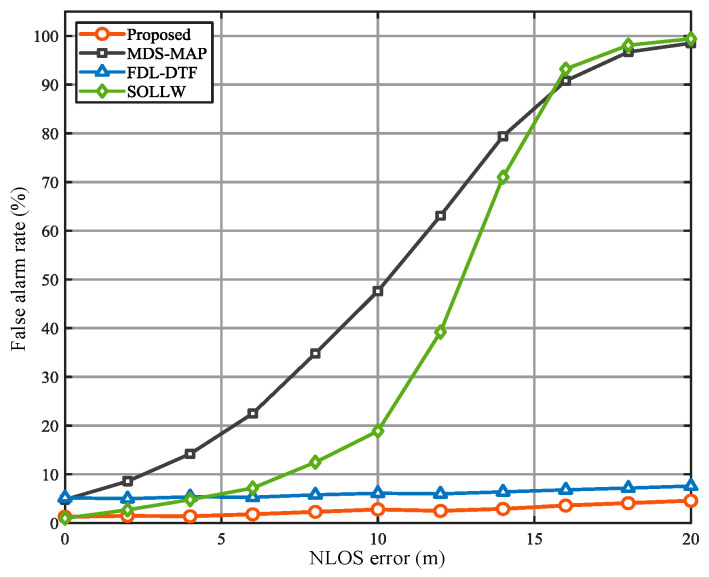
Variation in false alarm rate with increasing NLOS ranging error.

**Figure 10:**
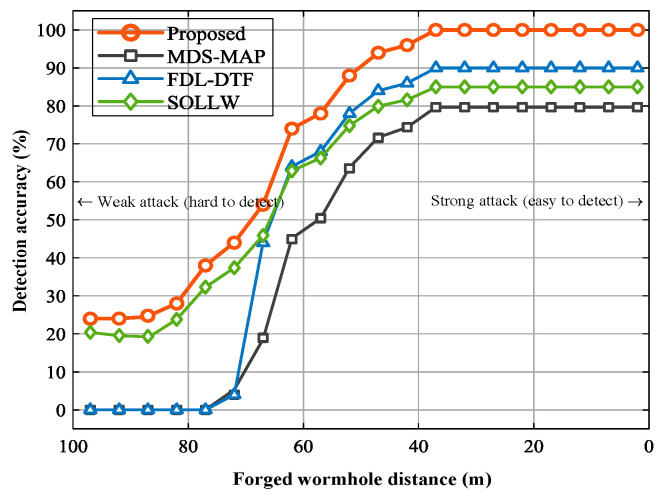
Comparison of detection accuracy of different algorithms under different wormhole attack intensities.

This subsection evaluates the performance of the proposed UWB-aware topology-stress defense mechanism under different NLOS error levels and wormhole attack intensities. The proposed method is compared with the classical geometry-based reconstruction method MDS-MAP [27], the federated-learning-based FDL-DTF method [10], and the one-class support vector machine-based SOLLW method [28]. The simulation results are shown in Figure 9 and Figure 10.

Figure 9 shows that the false alarm rates of MDS-MAP and SOLLW increase rapidly as the NLOS error increases, indicating their sensitivity to geometric distortion and noise variations. FDL-DTF exhibits better stability but still shows a gradual increase. In contrast, the proposed method maintains a low false alarm rate across all error levels because the adaptive stiffness model effectively reduces the contribution of unreliable NLOS links to topology stress.

Figure 10 shows the detection accuracy of different methods under varying wormhole attack intensities. When the attack intensity is low, MDS-MAP and FDL-DTF suffer from performance degradation due to their limited ability to capture weak geometric inconsistencies. In contrast, the proposed method maintains higher detection accuracy because topology stress can still capture residual geometric inconsistency even when forged distances are close to normal ranges. As attack intensity increases, all methods show improved detection performance, while the proposed method consistently achieves the best overall accuracy.

### 5.4. Experimental Validation

**Figure 11:**
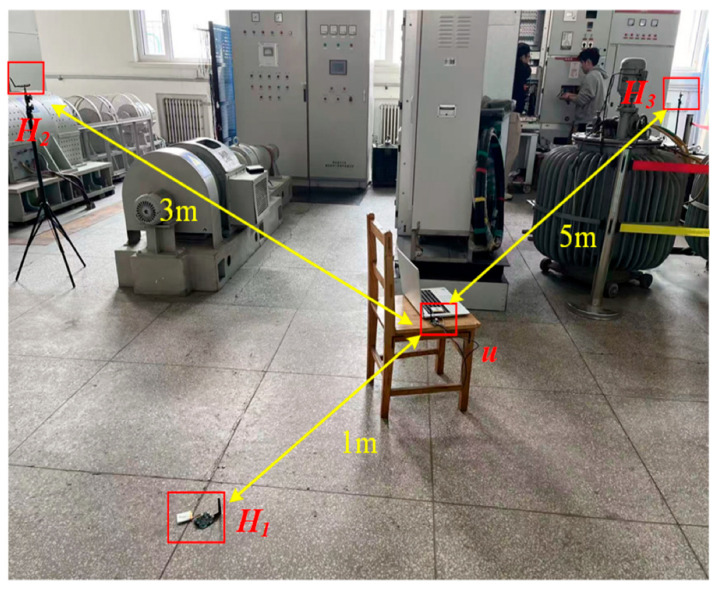
UWB hardware validation platform and test scenarios.

To verify the feasibility and practical effectiveness of the proposed wormhole attack defense mechanism under real UWB link conditions, a physical validation experiment is conducted using the UWB communication module developed in our previous work [29]. The experimental platform and test scenario are shown in Figure 11.

Three distance settings (1 m, 3 m, and 5 m) are considered, and both LOS and NLOS scenarios are constructed. In the LOS scenario, there is no obstruction between nodes, whereas in the NLOS scenario, a metallic obstacle is placed between the transmitter and receiver. The UWB module records the ranging value, first-path energy ratio, and CIR energy spread indicator. Table 3 summarizes the measured physical-layer statistics under different distances and propagation conditions.

As shown in Table 3, NLOS links exhibit larger ranging values, lower first-path energy ratios, and higher CIR spread indicators than LOS links across all tested distances, confirming that metallic obstruction introduces positive ranging bias and stronger multipath effects.

**Figure 12:**
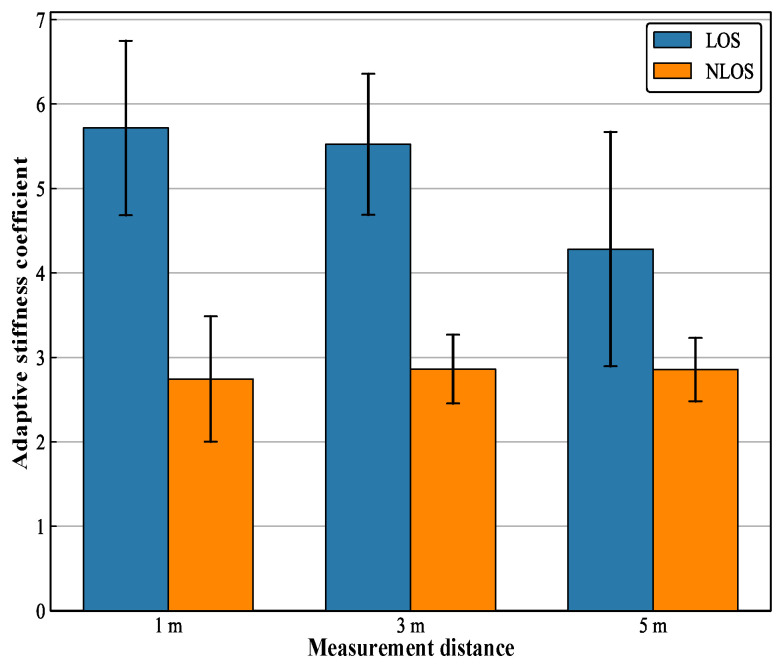
Comparison of adaptive stiffness coefficients under different distances and scenarios.

Based on these measurements, the adaptive stiffness coefficients in Figure 12 remain consistently higher for LOS links than for NLOS links at 1 m, 3 m, and 5 m, indicating that the proposed stiffness model can effectively quantify UWB link reliability under practical conditions.

**Figure 13:**
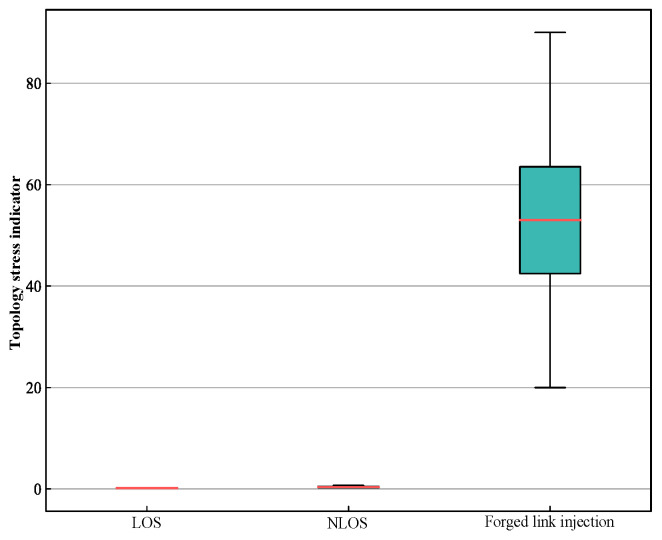
Comparison of topology stress indicators under different scenarios.

For topology verification, node *u* is taken as the reference center. Three normal LOS links at 1 m, 3 m, and 5 m are associated with neighboring nodes H1, H2, and H3 to construct a normal scenario. A mixed scenario containing NLOS links is used to represent environmental interference. In addition, the true physical distance between the malicious node W_2_ and node *u* is set to 8 m, while a forged observation distance of 2 m is injected to simulate a wormhole-induced adjacency relationship. The topology stress indicators under the three scenarios are shown in Figure 13. The boxplots illustrate the distribution and variability of topology stress under normal, NLOS-interference, and forged-link conditions. The average topology stress values are approximately 0.157, 0.390, and 66.620, respectively. This clear separation demonstrates that adaptive stiffness effectively suppresses NLOS-induced residual stress, while forged links introduce strong geometric inconsistencies and significantly increase topology stress.

**Figure 14:**
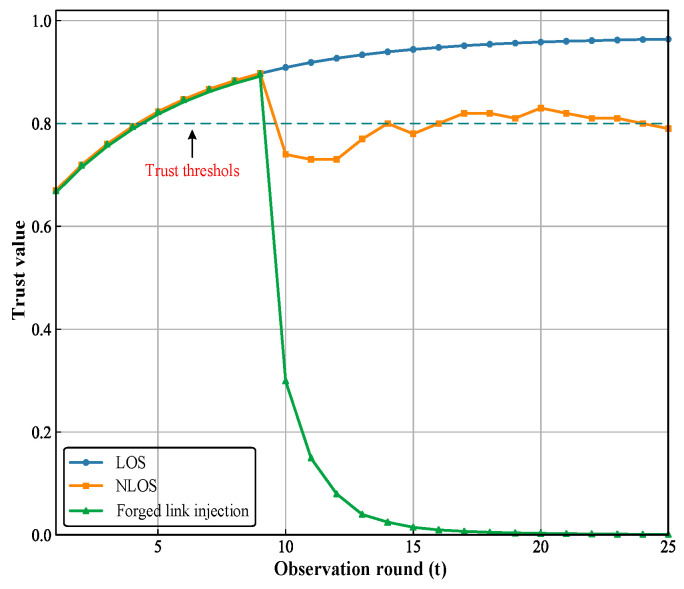
Trust evolution curves of nodes under different scenarios.

Figure 14 further shows that after 25 observation rounds, the trust value in the normal scenario increases to 0.963, whereas the forged-link scenario rapidly converges toward zero. The NLOS-interference scenario is penalized but not rejected, demonstrating that the temporal evidence fusion mechanism provides stable and robust decision-making under real UWB conditions.

Overall, the experimental results validate the complete defense pipeline of the proposed method. UWB physical-layer features are used to characterize link reliability and construct adaptive stiffness coefficients. The resulting topology stress effectively distinguishes environmental interference from forged adjacency relationships, while temporal trust fusion enables stable access decisions. These results confirm the applicability of the proposed method for wormhole attack defense in practical power wireless sensor networks.

## 6. Conclusions

To address the problem that wormhole attacks can forge long-distance adjacency relationships and undermine topology authenticity in power wireless sensor networks, this paper proposes a defense method that integrates UWB physical-layer awareness with topology stress analysis. The first-path power ratio and root-mean-square delay spread extracted from UWB channel impulse responses are used to characterize link propagation reliability, and adaptive stiffness modeling is introduced to mitigate the impact of complex propagation conditions on topology evaluation. Based on this, a topology stress indicator is constructed to capture local geometric distortion caused by forged adjacency relationships, and a Beta-evidence-based temporal fusion mechanism is designed to support reliable node access decisions. Simulation and hardware validation results demonstrate that the proposed method can effectively distinguish topology anomalies caused by environmental disturbances from those induced by wormhole attacks. It suppresses NLOS-induced false alarms while maintaining sensitivity to wormhole-induced topology distortion, enabling reliable detection of forged adjacency relationships in complex power environments. These results indicate that combining physical-layer propagation characteristics with topology consistency analysis provides an effective solution for topology security protection in power wireless sensor networks.

Although the proposed method shows promising performance in both simulation and hardware experiments, several limitations remain. This study mainly considers a single pair of colluding malicious nodes, while more complex multi-wormhole scenarios require further investigation. In addition, the hardware experiments are conducted in a controlled environment and have not yet been validated in large-scale real power deployment scenarios. Future work will focus on extending the method to more complex attack models and conducting field-level validation to further evaluate its practical applicability and long-term robustness.

## Figures and Tables

**Table 1:** Detailed channel-model parameters for LOS and NLOS scenarios.

Parameter	Symbol	LOS Scenario	NLOS Scenario
Channel fading model	Model	Rician Fading	Rayleigh Fading
Rice factor	*K_factor_*	10 dB	N/A(−∞)
Power-delay decay constant	Γ	5 ns	25 ns
Number of multipath components	*L_path_*	10–15	30–40
Maximum relative delay	$\tau_{max}$	30 ns	90 ns
First-path normalized gain	*G_FP_*	1.2	0.4
Doppler frequency shift	*f_d_*	0 Hz	0 Hz

**Table 2:** Simulation parameter settings.

Parameter	Symbol	Setting
Simulation area	Area	50 m × 50 m
Total number of nodes	*N*	20
Communication radius	*R*	20 m
Attack node locations	W_1_, W_2_	W_1_ near *u*, W_2_ near the gateway
Forged ranging value	*d*_attack_	3 m
Learning rate	α	0.003
Maximum number of iterations	*Step_max_*	400

**Table 3:** Statistical results of measured UWB physical-layer parameters under different distances and scenarios.

Scenario	Distance (m)	Mean Ranging Value (m)	Mean First-Path Energy Ratio (%)	Mean CIR Spread Indicator (ns)	Mean Stiffness Coefficient
LOS	1	1.141	41.10	6.552	5.717
NLOS	1	1.362	20.66	7.048	2.744
LOS	3	3.193	40.16	10.055	5.524
NLOS	3	3.417	23.43	10.501	2.862
LOS	5	5.177	35.39	9.331	4.282
NLOS	5	5.371	26.84	9.570	2.856

## Data Availability

The data presented in this study are available from the corresponding author upon reasonable request.
